# Human-built environment interactions: the relationship between subjective well-being and perceived neighborhood environment characteristics

**DOI:** 10.1038/s41598-022-25414-9

**Published:** 2022-12-17

**Authors:** Ali Reza Sadeghi, Maryam Ebadi, Fatemeh Shams, Sina Jangjoo

**Affiliations:** 1 Department of Urban Planning and Design, Faculty of Art and Architecture, Shiraz University, Shiraz, Iran; 2 Department of Urban Planning and Design, Faculty of Art and Architecture, Tarbiat Modares University, Tehran, Iran; 3 Askew School of Public Administration and Policy, College of Social Sciences and Public Policy, Florida State University, 768-2 California St., Tallahassee, FL 32304 USA

**Keywords:** Psychology and behaviour, Environmental impact

## Abstract

The neighborhood is one of the most fundamental urban elements and acts as the intermediary link between the city and citizens to enhance the quality of life. The present study examined the significance of the relationship between the subjective well-being of citizens and perceived neighborhood environment characteristics in urban historical fabrics for creating healthy neighborhoods. To this end, a survey research method was employed, and the data were collected via questionnaires. The population consisted of all the citizens of the historical neighborhood of Sange Siah in Shiraz, Iran, who lived or worked in the neighborhood and used the neighborhood spaces daily. A Nonparametric Spearman correlation coefficient was run to assess the correlation between the variables. The results showed that the component of social inclusion from among the six components of subjective well-being had a significant positive correlation with perceived neighborhood environment characteristics (r = 0.712). In the following, the components of satisfaction with life (0.614), mental well-being (0.569), positive and negative effect (0.526), and feeling of happiness (0.468) had a moderate positive correlation; and the component of physical and mental health also had a weak positive correlation with perceived neighborhood environment characteristics (0.230). In addition, the concept of subjective well-being with a correlation coefficient of 0.579 had a moderate positive correlation with perceived neighborhood environment characteristics, which indicates that the structural characteristics of the neighborhood have a significant relationship with the subjective well-being of the people living in the neighborhood.

## Introduction

Today, given the rapid growth of urbanization, living along with daily stresses has led to the increase of mental and psychological problems in humans. In such environments and for creation of healthy neighborhoods, the assignment of attention to the components of “physical and mental” health and the creation of “positive subjective well-being” among citizens has become a global challenge. This issue assumes importance when it is observed that the World Health Organization (WHO) has focused on building a network of healthy cities. Fitzpatrick believes that well-being is “a subjective component; so it relates to the feelings, perceptions, cognitions, and experiences of the same person that is the subject of well-being”. Fitzpatrick argues that no clear and complete definition of well-being can come into play and the question of “what is well-being” cannot be easily answered and evaluated^1^.

The London School of Economics and Political Science (LSE) considers health a product of people's positive subjective well-being. It is reported that many international organizations aim to improve and promote human well-being in urban environments^2^. In this regard, well-being in life is considered one of the most urgent innate desires and psychological needs of humans. In such a way, some researchers call happiness “subjective well-being”^3^, which refers to the extent to which one loves and enjoys his/her life^4^ or feeling supreme and relatively sustainable pleasures^5^. In fact, in this type of thinking, subjective well-being is shaped not only by having access to adequate food, water, and shelter but also through positive interactions with others, favorable physical and emotional experiences, avoidance of the sense of pain, and the ability to control conditions. Considering all these points, the primary sign of subjective well-being is that people believe they have at least a good life^6^. Nowadays, many researchers seek to investigate the dimensions of subjective well-being and the relationship between subjective well-being and its dimensions and components with individual, social, physical, and environmental issues such as physical and mental health, social inclusion, and built environment conditions. In the meantime, neighborhood environment characteristics, as the independent and external variables, play an essential role in predicting citizens’ subjective well-being^7^. A neighborhood is considered a significant territory and environment for people’s lives and enjoys a unique capability that affects citizens' well-being; besides, it is viewed as a desirable scale on which urban planners and designers can apply their desired changes^8^. However, there are only a limited number of studies investigating the significance of the relationship between neighborhood environment characteristics and their impacts on citizens' subjective well-being^7^.

Overall, it is necessary to have a comprehensive model of the dimensions of the “built environment” on the neighborhood scale and its effects on citizens’ subjective well-being. Such a model should be able to incorporate all the neighborhood environment characteristics and different aspects of subjective well-being^9^. The creation of positive subjective well-being in citizens is considered one of the main goals of life and an indicator of social sustainability^10^, which can be realized on various scales through planning and designing the built environments.

Considering the points mentioned above, the main purpose of the present study is to investigate the significance of the relationship between citizens’ subjective well-being and perceived neighborhood environment characteristics. Sange Siah Neighborhood, located in the historical fabric of the city of Shiraz in Fars Province in Iran, was selected as the sampling base and case study in order to conduct this research. The selected neighborhood has specific historical, physical, and social characteristics and is known as a historical “neighborhood” with a unique identity in Shiraz. Accordingly, the main question of this study can be arranged as follows: Is there any significant relationship between perceived neighborhood environment characteristics in urban historical fabrics and different dimensions of subjective well-being based on the citizens’ preferences? In this line, the research hypothesis is also formulated in this proposition that there seems to be a significant correlation between perceived neighborhood environment characteristics and the level of subjective well-being among citizens of historical fabrics. Therefore, in the following section, i.e., the literature review, the theories pertaining to subjective well-being and its relationship with neighborhood environment characteristics are reviewed and analyzed to explain the research's theoretical framework.

## Literature review

In the past two decades, various research has been conducted with different approaches to investigate the relationship between the characteristics of the built environment and subjective well-being. These research projects can be categorized into several general groups based on their chosen approach to this subject and research methodology. The first group is the research that has focused only on special and unique dimensions of subjective well-being with a reductionist approach and considered the concept of subjective well-being as equivalent and synonymous with a specific component. They have only analyzed this component or dimension to analyze subjective well-being. Some of the research includes 8 (emotional components as subjective well-being), 11 (individual physical health as subjective well-being), 14 (individual satisfaction as subjective well-being), 16 (mental health as subjective well-being), 17 (Physical and mental health as subjective well-being), 18 (individual satisfaction as subjective well-being), 19 (residential satisfaction and life satisfaction as subjective well-being). Although these research projects have tried to measure the relationship between the characteristics of the built environment and subjective well-being, they are seriously criticized due to the lack of a comprehensive approach to subjective well-being and its components and dimensions.

The second group is research that has tried to consider the multiple dimensions of subjective well-being with a holistic approach when examining the relationship or how the environment affects subjective well-being and has not focused on a specific aspect or dimension of subjective well-being. However, most of these research projects have examined subjective well-being in the form of two or three dimensions and have not been able to take a comprehensive and holistic view of subjective well-being and have not considered all aspects of this concept. Among these research projects, we can refer to research 12 (emphasis on the dimensions of vitality and mental health), 13 (depression and negative affective component), 15 (happiness and positive affective components), and 6 (positive and negative effective components, happiness and anxiety).

The third group is research that put the concept of subjective well-being together with different concepts and factors in their research with a simple look at the concept of subjective well-being and investigated the relationship of the built environment with several separate and different concepts simultaneously. Among these research projects, we can refer to 20, which measured the relationship between the built environment and several factors such as public transportation system, social deprivation, physical and mental health, and subjective well-being. The lack of serious focus on the concept of subjective well-being and its components and dimensions is a serious criticism of this group of research.

In this regard, Saelens et al.^11^, concluded that the Perceived neighborhood components (Land-use mix, Street connectivity, Accessibility, /aesthetics, and Residential Safety) play a significant role in the level of physical health as one of the components of mental well-being. Guite et al.^12^ concluded that three independent variables, the noise level of neighbors, feeling of overcrowding and density, and Fear of crime and harassment, affect people's subjective well-being. Mair et al.^13^ showed that the socio-economic status of people at the neighborhood level impacts people's subjective well-being. Lovejoy et al.^14^ showed that the Attractiveness of the neighborhood and the Safety perceived by the people living in the neighborhood as two important characteristics are more related to the component of People's satisfaction with the neighborhood in two types of traditional and suburban neighborhoods in Northern California.

Lyden et al.^15^ concluded that positive evaluation of subjective well-being is associated with important aspects of the built environment, including Accessibility to cultural amenities and public transportation. Bond et al.^16^ investigated the residential and environmental conditions of the neighborhood, on the level of mental health of people, that moving from apartments to villas increases the subjective well-being of the respondents, and in addition to the physical conditions of the housing, the variable of housing rent can also affect the subjective well-being of the residents. Friedman et al.^17^ showed that the Perceived neighborhood environment characteristics such as safety, social cohesion with the component of physical and mental health, and subjective well-being, in general, have a positive relationship. Ambrey & Fleming^18^, showed that public green spaces positively affect people's satisfaction with the neighborhood environment. Cao^19^, concluded that Street connectivity positively affects the evaluation of people's life satisfaction, while density negatively affects people's life satisfaction.

Ma et al.^20^, concluded that neighborhood density has negative effects on people's physical and mental health; while it has a positive effect on the subjective well-being of citizens, the perception of crime in a neighborhood is highly correlated with transportation and poor physical health. Also, the aesthetics of the neighborhood's structure and the characteristics of the neighborhood's social environment have stronger effects on the level of subjective well-being of the citizens than other characteristics of the neighborhood. Dong & Qin^8^, showed that among the objective characteristics of the built environment, only urban parks have a high correlation with the emotional components of subjective well-being; on the other hand, among the social characteristics of neighborhoods, only the relations between neighbors have a high correlation with subjective well-being. Kent et al.^6^ showed that perceived neighborhood environment characteristics strongly correlate with the perception of aesthetics and social cohesion of the neighborhood.

On the other hand, the view and approach to the environment, its components, and scale have also been different in the research. Some research projects have focused only on the social environment^13,16^, and another group has only considered the mental components of the environment as a criterion for action. Another group has only used the mental components of the environment as a criterion for action (11 and 17). Some research projects have only examined the objective components of the environment^12,14,15,20^. Also, a group of researchers has considered the environment as a concept consisting of objective and subjective components^6,19^ and objective, subjective and social components^8^. Regarding scale, a group of research has focused only on one type of environment, such as green space^18^. Another group examined the components and characteristics of the environment on the neighborhood scale^11,13,14,16,17,19,20^, and another group considered the environmental components on the city scale ^15,18^.

As it can be seen, the lack of attention to mental well-being as an integrated whole of various dimensions and components, as well as the lack of attention to the importance of citizens' perception of the components of the built environment, are the gaps in the research conducted to investigate the relationship between subjective well-being and neighborhood environment characteristics. Table 1 presents an overview of the studies conducted in this realm.

**Table 1:** Analysis of the studies conducted on significance of the relationship between neighborhood environment characteristics and subjective well-being of citizens.

Researchers	Variables related to neighborhood structural characteristics	Variables related to subjective well-being
Saelens et al. ^11^	Perceived neighborhood environment Walkability 1-Residential density 2-Land-use mix 3-Diversity 4-Accessibility 5-Street connectivity 6-Infrastructure and safety Neighborhood surroundings/aesthetics Residential Safety	The impact of perceived neighborhood environment such as walkability on the rate of increased physical activity was measured physical health of individuals as one of the components of Subjective well-being was measured
Guite et al. ^12^	Physical components of the environment 1-Design and maintenance 2-Noise (Street noise, Neighbor noise) 3-Density and escape 4-Fear of crime and harassment 5-Social participation	Evaluation of mental health as an effective factor on Subjective well-being was assessed -evaluation of vitality
Mair et al. ^13^	Structural characteristics of the neighborhood 1-neighbourhood socioeconomic and racial/ethnic composition 2-Neighborhood level services and facilities Measures of social processes 1-Social cohesion and ties with neighbor's 2-Perceived exposure to crime 3-Neighborhood disorder	The feeling of depression as a negative Affective component of Subjective well-being at the neighborhood level was assessed
Lovejoy et al. ^14^	Measures of neighborhood characteristics 1-Attractiveness 2-Quiet 3-Liveliness 4-Big yards 5-Safety 6-Mixed-use 7-Good infrastructure	The Neighborhood satisfaction as a cognitive component of Subjective well- being was assessed
Leyden et al. ^15^	Components evaluated at city scale 1-Accessibility 2-Aesthetics 3-Maintenance 4-Safety 5-Social capital	Happiness as a positive affective component of subjective well- being was assessed
Bond et al. ^16^	Evaluation of Perceived Neighborhood Environment 1-Attractiveness of buildings 2-Attractiveness of Neighborhood 3-Quiet and peaceful Neighborhood	Subjective well-being was assessed
Friedman et al. ^17^	Evaluation of Perceived Neighborhood Environment 1-Safety 2-Social cohesion 3-walkability of the neighborhood	-Physical and mental health as one of the dimensions of Subjective well-being was assessed
Ambrey & Fleming ^18^	Physical characteristics of the environment 1-public green space	The residents' life satisfaction as a cognitive component of Subjective well-being was assessed
Cao ^19^	Perceived neighborhood characteristics 1-Accessibility 2-Nuisance 3-Residential satisfaction -Neighborhood characteristics Objective 1-Population density 2-Land use mix 3-Cul-de-sacs density 4-Share of open space	The residents' satisfaction as a cognitive component of Subjective well-being was assessed
Ma et al. ^20^	Evaluation of perceived Neighborhood Environment 1-Residential density 2-Land-use mix 3-Diversity 4-Accessibility 5-Street connectivity 6-Infrastructure and safety 7-Neighborhood surroundings/aesthetics 8-Traffic hazards 9-Crime Evaluation of the neighborhood’s social environment: People in this neighborhood; 1-Are willing to help their neighbors 2-Can be trusted 3-Usually don’t get along 4-Do not share the same values	The components of mental and physical health, social exclusion, Transport disadvantage and satisfaction as a cognitive component of Subjective well-being and their correlation with each was assessed
Dong & Qin ^8^	Perceived neighborhood characteristics 1-Neighborhood safety 2-Residential convenience 3-Transit accessibility 4-Walkability/bikability Observed neighborhood characteristics 1-Land use density 2-Mixed land use 3-Block size 4-Distance to the nearest park Neighborhood social capital: my neighbors; 1-I know most of them 2-Get along in my neighborhood 3-Are willing to help each other	Positive affective components as one of the dimensions of positive Subjective well-being was assessed
Kent et al. ^6^	Perceived neighborhood environment Walkability 1-Residential density 2-Land-use mix 3-Diversity 4-Accessibility 5-Street connectivity 6-Infrastructure and safety Neighborhood appreciation 7-Neighborhood surroundings/aesthetics 8-Social cohesion Residential Safety 9-Traffic hazards 10-Crime Measure of the neighborhood’s social environment: my neighbors: 1-Are willing to help their neighbors 2-This is a close-knit neighborhood 3-Can be trusted 4-Generally don’t get along 5-Do not share the same values Neighborhood environment objective: Density of: 1-Population 2-4-way street intersections 3-Amenities and services 4-Train stations 5-Bus stops 6-Distance to park, river, ocean 7-Percentage of parkland use	The positive and negative effective components such as happiness and anxiety and life satisfaction of residents as the main dimensions of Subjective well-being

According to Table 1, only a few studies have examined the significance of the relationship between citizens’ subjective well-being in all its dimensions (physical and mental health, social inclusion, cognitive component, affective component) and perceived neighborhood environment characteristics, although numerous studies have assessed the effect of neighborhood environment characteristics on the subjective well-being of citizens. This point verifies the novelty and innovation aspect of this research.

Regarding what was mentioned above, it can be acknowledged that the “Neighborhood Environment Walkability Scale (NEWS)” is a useful scale that is utilized as a valid global scale to measure the neighborhood environment^11^. This scale consists of some of the dimensions and indicators required for assessing and measuring citizens’ perceptions of neighborhood environment characteristics, which are briefly referred to as “perceived evaluation of the neighborhood environment” in this article. These dimensions and indicators include residential density, land-use mix in neighborhoods, environmental diversity, different types of activities (stores, supermarkets, post offices, schools, fast foods, restaurants, and banks), accessibility (to mixed uses and types of public transportation), street connectivity, safety infrastructure for walking, safety across the neighborhood, aesthetic factors in the neighborhood (street trees and evaluation of buildings attractiveness), and traffic hazards and crimes^6^.

In sum, the main components of the perceived evaluation of the neighborhood environment are presented in Table 2.

**Table 2:** Explaining the main components of perceived evaluation of the neighborhood environment.

The main component	Sub-component	Definition	Sources
1-Walkability	Diversity	This sub-component shows the walking distance to all non-residential uses (restaurants, grocery stores, and other small retail stores, banks.)	^6,11,20^
	Accessibility	This sub-component refers to the accessibility to various shops, public transportation stations, and other uses	^6,8,11,15,19,20^
	Street Connectivity	This sub-component indicates that a neighborhood with much walkability with a mainly grid street pattern and the length of short blocks shows a more significant connection of the streets	^6,11,20^
	Safety Infrastructure for Walking	This component indicates the appropriate lighting level, optimal pavement, the strip separating the sidewalks from the passages, signs, and symptoms, and optimal maintenance	^6,11,14,15,20^
2-Neighborhood appreciation	Aesthetics	This sub-component shows the presence of street trees and views and evaluation of the attractiveness of buildings	^6,11,14,16,20^
	Social Cohesion	This sub-component response to statements such as People around my neighborhood are willing to help their neighbors and the existence of places for forming these collective behaviors	^6,13,17^
3-Residential Neighborhood safety	Residential Density	This sub-component shows the frequency of various neighborhood residences, from single-family detached homes to 13-story or higher apartments	^6,11,20^
	Crime Rate	This sub-component component examines the crime rate in the neighborhood and the lighting at night	^6,11,14,20^

**Figure 1:**
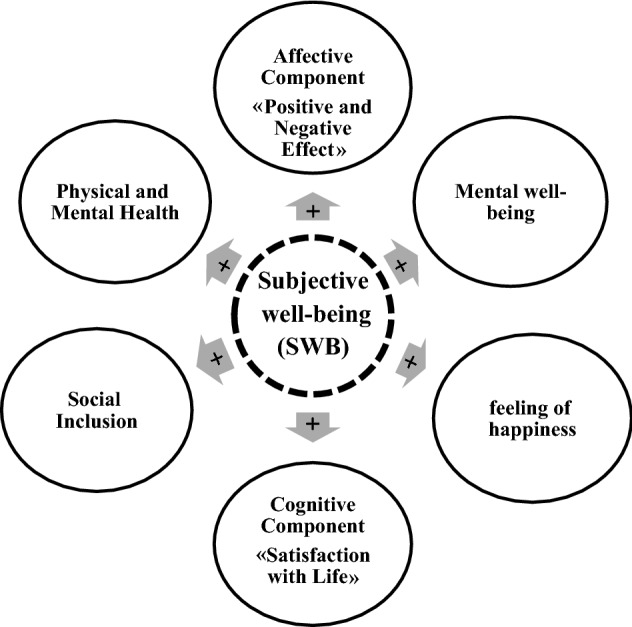
Main components of subjective well-being (SWB).

It was also stated that subjective well-being comprises all individuals' positive and negative evaluations of their lives. These include cognitive evaluation (satisfaction with life) and affective evaluation (positive and negative effects). Thus, “subjective well-being” can be regarded as an umbrella term for people’s various evaluations of their lives and events and generally refers to an evaluation of the body, mind, and conditions under which people live^21^. Although well-being and ill-being are subjective concepts, “subjective well-being” can be observed objectively in verbal and nonverbal behaviors and personal activities. It can be argued that the term well-being is often used interchangeably with subjective well-being^22^. In this definition, two aspects of subjective well-being are highly important and should be distinguished from each other. One is the cognitive dimension, usually perceived as individuals' satisfaction with life. At the same time, the other one is the affective dimension, usually considered as the feeling of happiness or discomfort in individuals or the balance between positive and negative effects^21^. According to the “Commission for the Measurement of Economic Performance and Social Progress (CMEPSP),” subjective well-being encompasses various aspects, such as a sense of happiness, cognitive evaluation including satisfaction with life, and affective evaluation including positive effects (e.g., pleasure and pride) and negative effects (e.g., stress, pain, and worry). In general, subjective well-being implies individuals’ evaluation of the inner well-being that they perceive they benefit from^23^. Russell believes subjective well-being refers to the person's perceptions of him/herself and subjective perception of his/her life experience. Subjective well-being encompasses individuals' affective and cognitive evaluations and presents a pleasant and advanced psychological state. It is a multidimensional concept that contains two broad areas, i.e., affective well-being and positive functioning. Affective well-being is a dimension of subjective well-being that includes perceptions of happiness, satisfaction with life, the balance of emotions, and positive and negative effects. Therefore, affective well-being includes a threefold structure: satisfaction with life, positive affect, and negative affect. As such, subjective well-being is defined by the definitions of affective well-being and positive functioning and includes elements of perceived happiness and satisfaction with life, the balance of positive and negative effects, psychological well-being, and social well-being. These elements are not unrelated to people’s daily behavior because positive and negative effects result from the factors that people apply in their lives and, thereby, assume great importance^24^. The main dimensions and components of subjective well-being are presented in Fig. 1 and Table 3 by analyzing and summarizing the findings of prior studies.

**Table 3:** Explaining the components consisting of citizens’ subjective well-being.

Component	Concept	Sources
Affective characteristics (positive and negative effects)	In fact, the concept of affective characteristics represents “positive affects” and “negative affects”. “Positive affects” result from positive reactions to activities (interest and engagement or participation in activities), while “negative affects” refer to one’s undesirable moods and affects and show people's negative responses to their own lives, health, events, and conditions	^21^
Satisfaction with Life	“Satisfaction with life” expresses the broad evaluation that a person has of his/her own life. The term life may point to all aspects of one’s life at a given time and is defined as a comprehensive judgment of one’s life from birth	^21,25^
Mental well-being	“Mental well-being” refers to a set of positive and negative thoughts and effects of individuals over the previous two weeks and is used to manage their thoughts and affects	^26^
Feeling of Happiness	“Feeling of happiness” refers to how people feel positively and pleasantly about their lives and is a multifaceted concept. Thus, due to the importance of happiness and its direct relationship with subjective well-being, the measurement of this feeling is necessary	^21,27^
Social Inclusion	“Social inclusion” is a multidimensional concept that includes physical aspects, logical aspects, social aspects (meeting and talking to friends and relatives, and the like), and so on. In addition, “social inclusion” results in the individual’s belonging to the society	^28^
Physical and Mental Health	The definition proposed by the World Health Organization about health in its preface of statute states that “health is the state of complete physical, mental, and social well-being and absence of illness and disability”. Therefore, it is one of the main components of subjective well-being	^20^

## Materials and methods

A descriptive-analytical research method in the form of a survey was employed to carry out the present study through library and field studies. All people living and working in the Sange Siah Neighborhood in the historical fabric of Shiraz city constituted this study's population. These individuals lived or worked in the neighborhood and had detailed knowledge of the neighborhood environment. Since the population's exact size was unknown, the sample size was calculated by the Cochran formula, which is used to determine the sample size when the population size is unknown. Based on this formula and with an error coefficient (d) of 0.1, the sample size was equal to 97.

**Figure 2:**
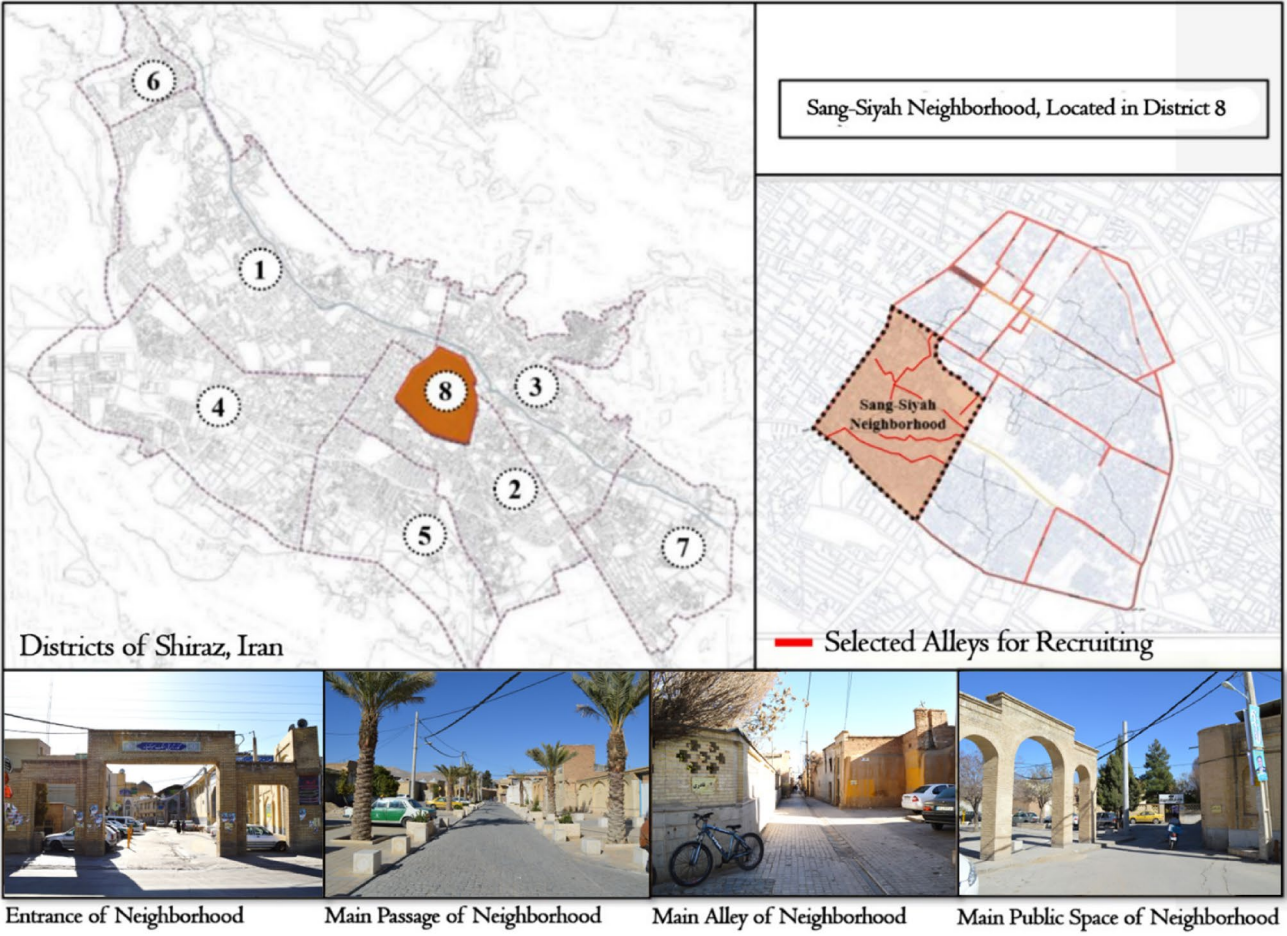
Location of the historical neighborhood of Sange Siah in historical fabric of Shiraz, Fars, Iran (Photos by: Sina Jangjoo).

Random sampling was used for the case study. For 21 days, from the beginning of July to August of 2021, researchers were continuously present at the passages and crossings of the neighborhood (esp. near the neighborhood's landmarks). They asked people over 18 years old who were crossing or doing some activity in public spaces (residents, passers-by, and vendors.) to fill out the questionnaire (inclusion criteria). For the researchers to have the most contact with the entire studied population, they were present in the neighborhood on working days and holidays and at different hours of the day and night. The authors asked some basic questions about whether the person is a resident of this neighborhood and whether they are willing to talk about the characteristics of their mental well-being. People who did not live in the neighborhood or did not want to express their feelings and perceptions of space were excluded from the questioning (exclusion criteria). Due to the high number of questions, the effort was to read them to the people so they would be more encouraged to answer. In the beginning, an explanation was given to the person about the purpose of the research and that there is no governmental affiliation. Figure 2 shows the Sange Siah Neighborhood's position in Shiraz in Iran. The passages where questioning took place and the most important elements of the neighborhood are marked in this image.

The historical neighborhood of Sange Siah is indeed one of the neighborhoods left from the Safaris and Atabakans era. The neighborhood can be divided into different areas based on the existing functional areas. Still, in general, it can be said that there is a favorable mix of residential and commercial land uses in the edges of the neighborhood, and in the inner part, religious and then commercial land uses prevail. Today, many of the landmark buildings in the area have become tourist attraction points, which not only attract a large number of people from inside and outside the country but also cause the economic and livelihood prosperity of the neighborhood and have reduced the deterioration process by encouraging other people to restore and renovate.

The central passage in the neighborhood, known as Sange Siah Passage, was once considered an important passage of the city that had formed important spaces and activities around it^29^. At present, this passage is also considered one of the most important passages of the old fabric of Shiraz city. In this passage, there are more than 30 known historic buildings and collections registered by Iran's Cultural Heritage Organization, and it also has a high potential in terms of values and physical and functional capacities that are rarely found in historical cities of Iran. Despite extensive destructions, the presence of elements, such as baths, mosques, and bazaars with a relatively active functioning near the residential fabric has rejuvenated the concept of “neighborhood” and “neighborhood center” in this part of Shiraz as it has been assigned meaning and identity.

Due to its morphological qualities and distinctive content richness, this neighborhood is considered a valuable area as a public space and contains cultural and environmental values. During the era, different directions of social life have been formed, and it has been the place of various urban events. However, in this neighborhood, in recent decades, along with the migration of authentic and native people from the neighborhood, it has accommodated different groups of immigrants with different immigration origins, norms, and cultural life patterns. Moreover, according to the current intra-group relations, they are influenced to different degrees by the culture of their origin society, which has made the neighborhood face managerial, economic, social, and physical problems. Therefore, paying attention to increasing the residents' subjective well-being is necessary, because this neighborhood has preserved the concept of "neighborhood" and "neighborhood center" due to its unique historical, physical, and social characteristics. Therefore, it has been chosen as a case study for this research, and it is known as a favorable scale for urban planners and designers can apply their desired changes.

Moreover, in order to investigate the relationship between citizens’ subjective well-being and the neighborhood environment characteristics in the study sample, a three-part questionnaire was developed based on a 5-point Likert scale (See Online Appendix 1). The first part included questions regarding the participants' demographic characteristics, such as gender, age, marital status, employment status, etc.

The second part encompassed questions relating to citizens’ perception of neighborhood environment characteristics, and the third part of the questionnaire consisted of the questions aiming at the measurement of subjective well-being components separately. The items related to subjective well-being indices are explained based on the measures introduced in Table 4. Each of the components that have been questioned has items that have been confirmed and validated in related research. Therefore, the questions of this survey questionnaire have all been adapted to global standards and localized to make it more accurate and easy for the respondents to understand.

**Table 4:** Measurement of the main components of citizens’ subjective well-being and its application in the research questionnaire.

Component	Concept			Sources
Affective characteristics (positive and negative effects)	In this study, a questionnaire, developed and introduced by Mroczek & Kolarz (1988), was employed in order to evaluate affective characteristics (positive and negative effects). After that, people like Joshanloo & et al. in the years (2016–2017) has proved the validity of the proposed factors and criteria. There were 12 items in the questionnaire where the first six ones were related to negative effects and emotions experienced by a person during the past month, and the remaining six items belonged to positive effects and emotions. In this regard, a Likert scale was used for scoring			^30^
Satisfaction with life	Satisfaction with Life Scale (SWLS) developed by Diener, Emmons, Larsen, and Griffin (1985). SWLS has been widely used and is a global assessment of Satisfaction with one’s life rather than with specific domains. It has shown strong internal reliability, and moderate temporal stability. Therefore, 5 items proposed as the standard items were used for the overall measurement of satisfaction with personal life. These 5 items include: “1-In most ways my life is close to my ideal; 2-The conditions of my life are excellent; 3-I am satisfied with my life; 4-So far, I have gotten the important things I want; 5-If I could live my life over, I would change almost nothing;”			^20,25^
Mental well-being	1-The Warwick-Edinburgh Mental Wellbeing Scale (WEMWBS) 1 is a validated measure of mental wellbeing that has been used nationally, regionally and locally and seen as an effective tool There is a 14-item and a 7-item (WEMWBS) questionnaire that produces a single score. It is self-completed (for people aged 13 +) to record ‘statements about their thoughts and feelings over the past two weeks. The questionnaire validated by the University of Edinburgh In the present study, only the first 7 items expressing positive thoughts and feelings were evaluated due to the correlation between individuals' positive thoughts and emotions and increased levels of subjective well-being. These 7 items are as follows: “1-Feeling optimistic about the future 2-Feeling useful 3-Feeling relaxed 4-Dealing with problems well 5-Thinking clearly 6-Feeling close to other people Able to make up my own mind about things”			^26^
Feeling of happiness	According to the study Lyubomirsky & Lepper, (1999) the Subjective Happiness Scale were derived from the literature and have demonstrated reliability and validity. “Feeling of happiness” was measured by the 4 items presented below: “1-I consider myself: not a very happy or unhappy person 2-Compared to most of my peers, I consider myself: less happy or more happy 3-Some people are generally very happy. They enjoy life regardless of what is going on. To what extent does this characterization describe you? 4-Some people are generally not very happy. Although they are not depressed, they never seem as happy as they might be. To what extend does this characterization describe you?”			^27^
Social inclusion	Based on the reviewed literature to measure the level of social inclusion with other people in the neighborhood, the following items were used: 1-Income 2-Employment status 3-Social support from friends, family and etc 4-Participation in social activities			^28,31^
Physical and mental health	In this study and research related to the research approach, to measure individuals’ physical and mental health, as one of the main components of subjective well-being, a questionnaire called SF-12 was used. It contains 12 items and has been proved to be a valid evaluation tool in this field in the United States and other countries			^20^
Perceived evaluation of the neighborhood environment	Walkability	Diversity	1-I believe that a diversity of activities such as (store, supermarket, post office, school, fast food, restaurant, bank, etc.) can be seen in this neighborhood	^6,8,11,13–17,19,20^
		Accessibility	2-I believe that in this neighborhood, I can easily access public transportation such as (subway, bus, taxi…) 3-I believe that the distance between my place of residence and my workplace is suitable	
		Street Connectivity	4-I believe that in this neighborhood the streets are connected to each other and can easily walk from one street to another	
		Safety Infrastructure for Walking	5-I believe that the sidewalks in this neighborhood have desirable Pavement 6-I believe that the lighting is properly provided in this neighborhood 7-I believe that there is enough furniture for people to sit in this neighborhood 8-I believe there are many green and open spaces in this neighborhood	
	Neighborhood appreciation	Aesthetics	9-I believe that the form of buildings in this neighborhood is attractive and beautiful	
		Social Cohesion	10-I believe that there are places for people's social cohesion in this neighborhood (parks, green spaces, cultural centers, etc.)	
	Residential Neighborhood safety	Residential Density	11-I believe that the number of residential buildings in this neighborhood is balanced	
		Crime rate	12-I believe that there is not much crime in this neighborhood	

SPSS was used for data analysis. Also, the content validity of the questionnaire was determined by experts in urban design and psychology; and Cronbach’s alpha method was used to assess its reliability. The qualitative content validity method was used to assess the validity of the research. In this regard, three experts in urban design and two in psychology were asked to determine whether the questionnaire measured all the critical aspects of citizens’ subjective well-being and the neighborhood environment characteristics. Also, are the questions formulated correctly to represent the indicators in question? The results of this study showed that the questionnaire has the necessary validity.

**Figure 3:**
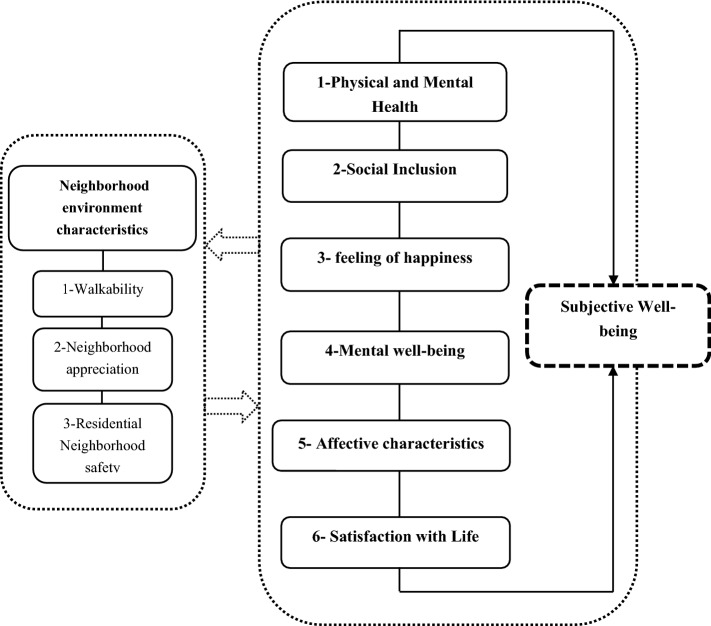
The conceptual framework and the relationship between neighborhood environment characteristics and major dimensions of subjective well-being.

Accordingly, Cronbach’s alpha coefficient of 0.780 was obtained for all the items, indicating that the variables' data are sufficiently reliable. The Kolmogorov–Smirnov test evaluated the normality of data distribution, and the value of 0.002 was obtained. As it is smaller than 0.05, the data are not within the normal range. Therefore, the correlation between the variables was examined using the nonparametric Spearman correlation test. Figure 3 presents the conceptual framework and the perceived relationship between the research variables.

### Ethics approval and consent to participate

The study was approved by *Shiraz University Faculty of Art and Architecture Ethics Committee* and performed in accordance with the *World Medical Association Declaration of Helsinki* for ethical principles for medical research involving human subjects.

All procedures performed in studies involving human participants were in accordance with the ethical standards of the institution or practice at which the studies were conducted. Also, it is important to clarify that this article does not contain any studies involving animals performed by any of the authors; All procedures performed in studies involving human participants were in accordance with the ethical standards of the institutional and/or national research committee and with the *1964 Helsinki Declaration* and its later amendments or comparable ethical standards; And *informed consent* was obtained from all individual participants (subjects) involved in the study.

## Results

Concerning the demographic information of the sample group, the results showed that 76% of the participants were male, and the remaining 24% were female. In addition, 28% of the respondents were placed in the 18–25-year age group, 37% in the 26–35-year age group, 21% in the 36–45-year age group, 9% in the 46–55-year age group, 3% in the 56–65-year age group, and 1% were over 65 years of age. Regarding marital status, 49% were single, 50% married, and 1% were divorced. Regarding the educational level, 38% of the respondents held an educational degree lower than high school, 40% had a high school diploma or an Associate degree, 11% had a bachelor’s degree, and 11% had a master’s degree. Considering the employment status, 31% of the respondents were unemployed or had a non-permanent job, 28% had a fixed-time job with a fixed payment, 22% had a fixed-time job with variable payment, 11% were homemakers, 3% were retired, and 5% were university students. Also, 6% of the respondents lived alone, and 94% lived with their families. In terms of income level, 40% of the respondents had a household income of fewer than 240 dollars; 37% had an income of 240–480 dollars; 16% had an income of 480–960 dollars, and 4% had an income higher than 960 dollars per month (See Online Appendix 2).

As stated above, the present study's main purpose was to examine the significance of the relationship between the six components of subjective well-being and perceived neighborhood environment characteristics. It is noteworthy that perceived neighborhood environment characteristics consist of three general components, namely.

1. Walkability (sub-components of diversity, accessibility, street connectivity, and safety infrastructure for walking),
2. Neighborhood appreciation (including sub-components of aesthetics and social cohesion), and.
3. Residential neighborhood safety (including sub-components of residential density and crime rate).

Nonparametric Spearman correlation coefficient was run to assess the correlation between the variables (data was not normally distributed evaluated with the Kolmogorov-Smirnoff-Test). The results of examining the relationship of the components of subjective well-being with the three components of perceived neighborhood environment characteristics are summarized in Tables 5, 6, 7 separately. Table 8 also shows the correlation between the sum of perceived neighborhood environment characteristics and the constituent components of citizens’ subjective well-being. In Tables 5, 6, 7, 8, the value of N = 97 and the value of the critical interval for significant rho value was zero.

**Table 5:** The correlation of walkability and its sub-components with constituent components of citizens’ subjective well-being.

Total subjective well-being	Physical and mental health	Social inclusion	Feeling of happiness	Mental well-being	Satisfaction with life	Affective characteristics	Spearman's rho	Sub-Component	Component
0.515	0.471	0.377	0.426	0.532	0.483	0.484	Correlation Coefficient	Diversity	Walkability
0.616	0.553	0.415	0.533	0.622	0.546	0.551	Correlation Coefficient	Accessibility	
0.428	0.383	0.319	0.350	0.501	0.342	0.409	Correlation Coefficient	Street connectivity	
0.326	0.204	0.420	0.253	0.286	0.423	0.336	Correlation Coefficient	safety infrastructure for walking	
0.619	0.477	0.509	0.499	0.618	0.606	0.573	Correlation Coefficient	Walkability

**Table 6:** The correlation of neighborhood appreciation and its sub-components with the constituent components of citizens’ subjective well-being.

Component	Sub-Component	Spearman's rho	Affective characteristics	Satisfaction with life	Mental well-being	Feeling of happiness	Social inclusion	Physical and mental health	Total subjective well-being
Neighborhood appreciation	Aesthetics	Correlation Coefficient	0.347	0.491	0.281	0.257	0.458	0.277	0.363
	Social cohesion	Correlation Coefficient	0.373	0.534	0.313	0.336	0.486	0.284	0.405
	Neighborhood appreciation	Correlation Coefficient	0.360	0.511	0.302	0.289	0.477	0.283	0.385

**Table 7:** The correlation of residential neighborhood safety and its sub-components with the constituent components of citizens’ subjective well-being.

Total subjective well-being	Physical and mental health	Social inclusion	Feeling of happiness	Mental well-being	Satisfaction with life	Affective characteristics	Spearman's rho	Sub-Component	Component
0.412	0.459	0.362	0.378	0.471	0.360	0.328	Correlation Coefficient	Residential density	Residential Neighborhood safety
0.202	0.388	0.249	0.381	0.396	0.514	0.381	Correlation Coefficient	Crime rate	
0.482	0.419	0.426	0.440	0.521	0.510	0.413	Correlation Coefficient	Residential Neighborhood safety

**Table 8:** The correlation between neighborhood characteristics and components of citizens’ subjective well-being.

Total subjective well-being	Physical and mental health	Social inclusion	Feeling of happiness	Mental well-being	Satisfaction with life	Affective characteristics	Spearman's rho	Component
0.619	0.477	0.509	0.499	0.618	0.606	0.573	Correlation Coefficient	Walkability
0.385	0.283	0.481	0.289	0.302	0.511	0.360	Correlation Coefficient	Neighborhood appreciation
0.482	0.419	0.426	0.440	0.521	0.510	0.413	Correlation Coefficient	Residential Neighborhood safety
0.579	0.230	0.712	0.458	0.569	0.614	0.526	Correlation Coefficient	perceived neighborhood environment characteristic

The relationship of walkability as the first perceived neighborhood environment characteristic and its sub-components with components of subjective well-being was examined and it was revealed that the sub-components of diversity, accessibility, and street connectivity had a moderate positive correlation with the components of citizen’s subjective well-being (correlation coefficient between 0.377 and 0.622). Meanwhile, there was a weak positive correlation between the sub-component of street connectivity and social inclusion (0.319). Similarly, there was a slight positive correlation between the sub-component of safety infrastructure for walking and the components of citizens’ subjective well-being (0.326).

In the same way, the relationship of the neighborhood appreciation as the second perceived neighborhood environment characteristic and its sub-components with the components of subjective well-being was assessed. The results showed that the sub-components of aesthetics and social cohesion had a moderate positive correlation with the constituent components of citizen’s subjective well-being (0.363 and 0.405, respectively). This is so while the correlation between the sub-component of aesthetics and the components of mental well-being (0.281), feeling of happiness (0.257), and physical and mental health (0.277) is positive and weak. In addition, there was a slight positive correlation between the sub-component of social cohesion and the components of mental well-being (0.313) and physical and mental health (0.284).

In addition, the relationship of residential neighborhood safety as the third perceived neighborhood environment characteristic and its sub-components with the components of subjective well-being was examined. It was observed that there was a moderate positive correlation between the sub-component of residential density and the components of citizens’ subjective well-being (0.412). This is so while there is a weak positive correlation between this sub-component and the component of affective characteristics (0.328). Furthermore, the sub-component of crime rate had a weak positive correlation with the components citizens’ subjective well-being (0.202). However, only the component of satisfaction with life from among the components of subjective well-being had a relatively higher correlation with crime rate (0.514).

**Figure 4:**
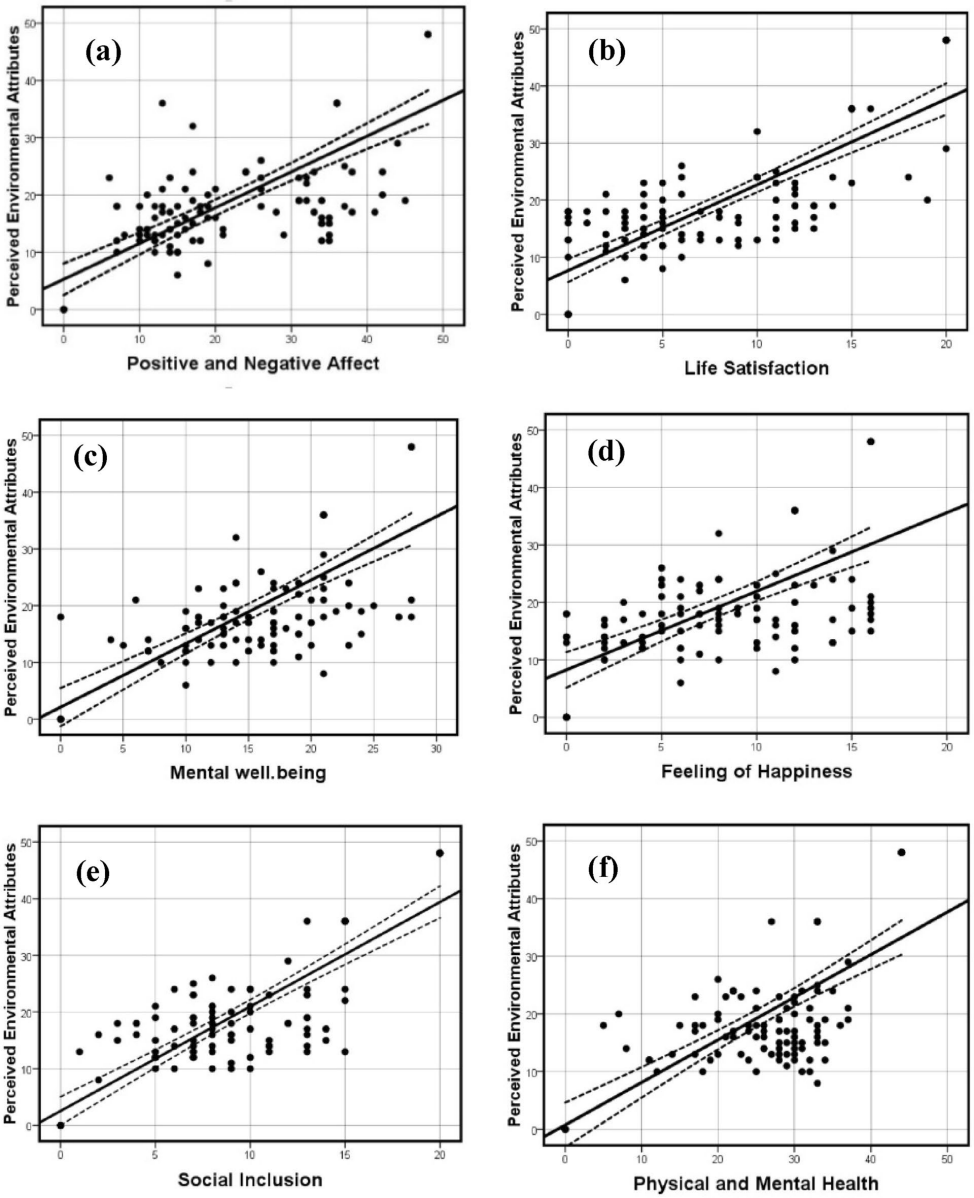
The scatter graphs illustrating the correlation between perceived neighborhood environment characteristics and factors of citizens' subjective well-being.

According to the results of data analysis in Table 8, it can be argued that only the component of social inclusion from among the six components of subjective well-being has a strong correlation (0.712) with perceived neighborhood environment characteristics. In the next ranks, the components of satisfaction with life (0.614), mental well-being (0.569), affective characteristics (0.526), ​​and feeling of happiness (0.458) are respectively placed that have a moderate correlation with perceived neighborhood environment characteristics. Finally, the component of physical and mental health had a weak correlation with perceived neighborhood environment characteristics (0.230). Overall, it can be claimed that the concept of subjective well-being has a moderate correlation (0.579) with perceived neighborhood environment characteristics. The scatter graphs, obtained using Spearman's rank correlation test, allow the visualization of bivariate data. In each of these graphs, the vertical axis shows the value of perceived neighborhood environment characteristics, and the horizontal axis shows the value of different factors of citizens' subjective well-being (See Fig. 4). The followings illustrate the existence of positive and medium correlations between the two variables in graphs (a), (b), (c), and (d); an almost strong positive correlation between the two variables in graph (e); and a weak positive correlation between the two variables in graph (f).

## Discussion

In general, the findings of this study suggest that perceived neighborhood environment characteristics are significantly related to different aspects of subjective well-being and have a significant positive correlation. According to the results in Tables 5, 6, 7, 8, it was found that the component of “social inclusion” from among the components of citizens’ subjective well-being had the highest positive correlation with perceived neighborhood environment characteristics. This finding is consistent with the results of the studies carried out by Lovejoy et al.^14^, Ma et al.^20^, and Dong and Qin^8^. In general, urban neighborhoods play a significant role in people’s lives, and, thereby, they are of great importance in the basic occasions of people and in establishing social relationships. The existence of “walkable” neighborhoods provides optimal physical opportunities to pause and reflect more on the neighborhood environment and, accordingly, access to the neighborhood's public spaces and its functional elements will increase. This important point can provide the foundation for people’s proper social communications within the neighborhood. In this way, urban neighborhoods with high walkability provide a suitable environment for the movement and pause of pedestrians and can increase social inclusion. “Neighborhood appreciation” also enhances social inclusion so that the apparent beauty of the neighborhood and one's decision to be present in the neighborhood environment can enhance residents’ perceptions of the neighborhood and its spaces and promote social inclusion among the residents by increasing social cohesion. Thus, the presence of spaces with apparent beauty, social cohesion, and environmental safety in the neighborhood results in the higher attendance of people in public spaces of the neighborhood at different times of the day (especially for women^32^) and also leads to the increased social inclusion among the individuals. Similarly, in the current neighborhood under study (Sange Siyah), the existence of public spaces, such as a beautiful mosque and marketplace beside the residential fabric, a continuous walkable path, a strong neighborhood center, safe spaces for gatherings and passage of leisure time, and social events and various religious rituals in the neighborhood has led to the creation of strong perceptions and a sense of belonging among the residents. Physical and social diversity, walkable spaces, and different public areas in the neighborhood have increased social inclusion in the studied neighborhood.

Moreover, the “satisfaction with life” component had a moderate positive correlation with perceived neighborhood environment characteristics. Indeed, satisfaction with life refers to one's broad evaluation and comprehensive judgment of his/her life from birth. This result is consistent with that of the study conducted by Cao^19^ because the concept of this component has a semantic overlap with the definition of the residential satisfaction component, where Cao defines it as the extent to which the neighborhood characteristics meet the current needs of families. In fact, according to the results of this study, the existence of neighborhoods with a high degree of walkability leads to an increased level of overall citizen satisfaction and, consequently, the increased level of residents’ subjective well-being and health. In addition, the promoted praise from the neighborhood because of its impact on people’s perceptions of physical beauty and social cohesion of the neighborhood is important in increasing the overall satisfaction with life. This is so because “satisfaction with life” includes reasonable personal evaluations that the residents have about the environment and structure of their neighborhood; therefore, it greatly affects the residents' subjective well-being.

People with different religions, income levels, occupations, and cultural characteristics live and work in the Sange Siyah neighborhood. It seems that the existence of these differences and the neighborhood's need to strengthen walkability, residential neighborhood safety, and neighborhood appreciation has caused that, from the respondents' point of view, the "satisfaction with life" component had a moderate positive correlation with perceived neighborhood environment characteristics.

The “mental well-being” component had a moderate positive correlation with perceived neighborhood environment characteristics. Mental well-being represents a set of all evaluations (positive and negative) that individuals have of their lives and indicates their positive and negative thoughts and feelings. This finding is in line with the results obtained by Dong and Qin^8^. Moreover, the results obtained in the studies conducted by Mair et al.^13^, Leyden et al.^15^, Dong and Qin^8^, and Kent et al.^6^ confirm the availability of the correlation between the “affective characteristics” component of subjective well-being and neighborhood environment characteristics. Their findings are consistent with the present study's results, asserting a significant positive relationship and a moderate correlation between “affective characteristics” as individuals' positive and negative effects and perceived neighborhood environment characteristics. On the other hand, the significant positive correlation of “feeling of happiness,” which is a kind of evaluation of positive emotions and moods, lack of depression, and lack of anxiety, with perceived neighborhood environment characteristics is consistent with the research findings reported by Leyden et al.^15^ and Kent et al.^6^.

There are safe pathways, historical monuments, and beautiful and attractive public spaces in Sange Siyah which bring joy and happiness to see them. However, in this same neighborhood, there are buildings with physical deterioration and abandoned public spaces, as well as unsafe and crime-prone alleys that create a sense of worry and anxiety in a person. It seems that what was mentioned caused that, from the respondents' point of view, mental well-being and feeling of happiness had a moderate positive correlation with perceived neighborhood environment characteristics.

The studies conducted by Bond et al.^16^ and Ma et al.^20^ confirm the existence of the correlation between the component of “physical and mental health” and perceived neighborhood environment characteristics, which is also supported by the results of the present study. It should be mentioned that physical and mental health is defined as the state of complete physical, mental, and social well-being and a lack of illness and disability in individuals. The present study showed that the component of “physical and mental health” from among the components of citizens’ subjective well-being being significantly correlated with perceived neighborhood environment characteristics had the least level of positive correlation with perceived neighborhood environment characteristics.

The male respondents in the Sange Siyah neighborhood were mainly young and middle-aged. It seems that these people have faced fewer problems related to physical and mental health due to their gender and age characteristics. In this sense, this component had the least level of positive correlation with perceived neighborhood environment characteristics.

Subjective well-being is one of the essential goals in achieving social sustainability, and, thereby, it is of paramount importance in human life. Considering this study's findings and the correlation between different dimensions of subjective well-being and perceived neighborhood environment characteristics, some strategies have been presented in Table 9 for enhancing citizens’ subjective well-being by increasing the quality of neighborhood environment characteristics. It should be noted that these strategies are mentioned according to the results obtained from the present research. It was determined that among the six components of subjective well-being with perceived neighborhood environment characteristics, in order, the component of Social Inclusion with positive and strong correlation at the first level, the component of Satisfaction with Life at the second level, Affective Component (positive and negative effect) at the third level, the Feeling of Happiness at the fourth level with a positive and weak correlation, and therefore the component of Physical and Mental Health is on the fifth level with a positive and weak correlation. Therefore, according to table number 8, an effort has been made to increase the level of subjective well-being of the residents of Sange Siah neighborhood based on the research results obtained and considering the significance level of each of the perceived components of the neighborhood in relation to the components of Subjective well-Being of the residents, strategies in 5 levels and in order of the degree of significance and correlation (strong-moderate-weak) of the components of subjective well-being with the perceived neighborhood environment characteristics (in order of priority and degree of significance) to increase the Subjective well-being of the citizens of this neighborhood as much as possible to be presented. In this way, it was determined that among the three components of Walkability, Neighborhood appreciation, and Residential Neighborhood Safety, the priority of improving Walkability, Residential neighborhood safety, and Neighborhood Appreciation are important. The components of subjective well-being (Social Inclusion, Satisfaction with Life, Affective Component, the Feeling of Happiness, and Physical and Mental Health) in the first level were more correlated with Walkability (0.619), and in the second level with the Residential neighborhood safety, (0.482) and the last level with the Neighborhood appreciation (0.385). Therefore, the strategies were made to increase subjective well-being according to the priority of each perceived environmental component.

**Table 9:** Goals and strategies for promoting citizens’ subjective well-being in neighborhood as an artificial environment.

Strategy			Goal	Priority
Neighborhood Appreciation (Third priority)	Residential Neighborhood Safety (Second priority)	Walkability (First Priority)		
Improvement of the neighborhood's physical condition Through creative design in the facade of buildings, pavements	The use of lighting suitable for the movement of pedestrians along the street at night (night lighting)	Creating diverse uses along the path for the presence of different social groups Creating spaces for social gatherings and the activity of peddlers and public arts in the neighborhood diversity in functions in such a way that it attracts different social groups, ages, etc -Continuity of footpaths from origin to destination	Promotion of social inclusion across the neighborhood	First level
Non-use of solid and integrated materials such as asphalt and concrete in place on the pavement	Create active users and day and night to minimize soundless urban bodies Avoiding the placement of Coarse-grained and rigid buildings on the edge of the main routes in the neighborhood	Creating a neighborhood park as a green element in combination with the pedestrian network Easy accessibility to all types of public transportation (Subway, bus, bike, etc.) and services needed by citizens on a walking scale Creating of bicycle lanes in the neighborhood for public use	Increasing overall satisfaction in the neighborhood	Second level
Improving the quality of sensory richness and the feeling of relaxation and pleasantness towards the space by using the element of water and vegetation	Absence of indefensible spaces such as dark corners, wastelands and abandoned buildings	Enhancing the quality of excitement by holding street shows and competitions at the neighborhood level Creating purposeful cultural-recreational collective hangouts on the way in the neighborhood	Promotion of affective characteristics and positive effects in citizens	Third level
Creating aesthetic elements (statues, flowerpots, decorative trees and plants, fountains, lighting, traffic lights, trash cans, benches, bus station canopy, public telephone booth, mailbox, etc.)	Increasing safety by activities such as, Pharmacy, clinic and telephone taxi in different parts of the neighborhood Increasing safety by using transparent bodies and creating windows facing the street in buildings	Creating a context for the establishment of galleries, museums, handicraft exhibitions, etc Creating wide sidewalks next to riding routes and equipping it with special facilities (suitable materials for pavements) holding ceremonies and street parties in the neighborhood	Increasing feeling of happiness in individuals across the neighborhood	Fourth level
Using creeping plants and combining it with the element of water to create visual pleasantness along the path in the neighborhood	Controlling the entry of cars into the street to reduce traffic and increase pedestrian safety Creating lanes for bicycles and disabled people	Developing and promoting sports, especially public sports, performing morning sports in the park and green spaces at the neighborhood Expansion of comfort facilities and outdoor spaces in the neighborhood Increasing support programs and welfare, educational and health facilities	Increasing citizens’ physical and mental health across the neighborhood	Fifth level

## Conclusion

**Figure 5:**
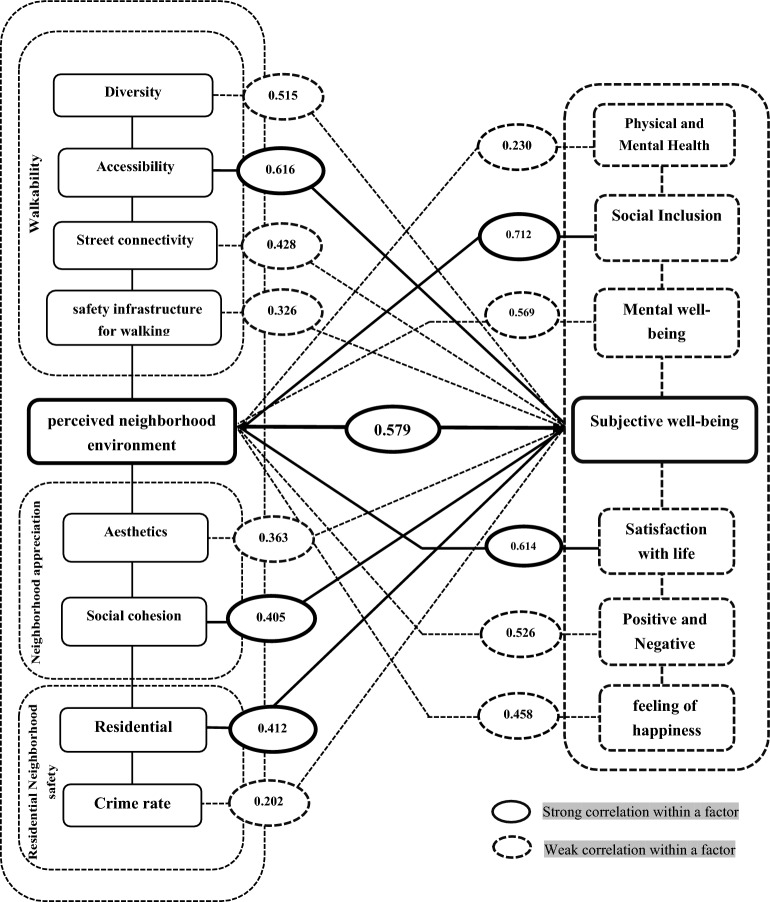
Correlation between perceived neighborhood environment characteristics and factors of citizens' subjective well-being.

The main purpose of the present study was to examine the significance of the relationship between citizens’ subjective well-being and perceived neighborhood environment characteristics for creation of healthy neighborhoods. Hence, a systematic review of the previous literature was first carried out in order to respond to the main research question and validate the research hypothesis (Table 1) and, then, the theoretical foundations related to the research keywords were reviewed. The results of this review focused on explaining the dimensions, components, and indicators pertaining to the measurement of perceived neighborhood environment characteristics (Table 2) and citizens’ subjective well-being (Table 3, Table 4, and Fig. 1). In the next step, and after examining the reliability and validity of the research questionnaire and collecting the data from the sample group, the correlations between the variables were analyzed using Spearman correlation test (Tables 5, 6, 7, 8). The results showed that the main hypothesis of the study is accepted (Fig. 5). In fact, it had been hypothesized that there is a significant correlation between perceived neighborhood environment characteristics (with components of: walkability, neighborhood appreciation, and residential neighborhood safety) and citizens’ subjective well-being (components of: social inclusion, satisfaction with life, mental well-being, affective characteristics, feeling of happiness, physical and mental health) in historical fabrics.

According to Figure number 5, the results of this research show that among the perceived neighborhood environment characteristics, accessibility (0.616) has a positive and strong correlation, social cohesion (0.405), and residential density (0.412) have a positive and moderate correlation with subjective well-being. Also, among the six components of subjective well-being, the component of social inclusion had a significant positive correlation with perceived neighborhood environment characteristics (r = 0.712). In the following, the components of satisfaction with life (0.614), mental well-being (0.569), positive and negative effect (0.526), and feeling of happiness (0.468) had a moderate positive correlation; and the component of physical and mental health also had a weak positive correlation with perceived neighborhood environment characteristics (0.230). In addition, the concept of subjective well-being with a correlation coefficient of (0.579) had a moderate positive correlation with perceived neighborhood environment characteristics, which indicates that the structural characteristics of the neighborhood have a significant relationship with the subjective well-being of the people living in the neighborhood. Therefore, according to the review of previous studies, it was found that previous related researchers have considered the effect of specific aspects of the built environment on a limited number of subjective well-being components; but in the meantime, there is a gap in the literature related to exploring the effects of the built environment on multiple components of subjective well-being. Thus, this study tried to fill this gap. It analyzed the relationship between the perceived variables of the built environment with multiple elements of subjective well-being to show a more comprehensive picture of the perceived neighborhood environment characteristics on subjective well-being. It is hoped that the findings of this study, especially the strategies presented in Table 9, can provide the grounds for the promotion of citizens’ subjective well-being by enhancing the quality of neighborhood environment characteristics.

## Supplementary Information

Supplementary Information 1.

Supplementary Information 2.

## Data Availability

The datasets used and/or analyzed during the current study available from the corresponding author on reasonable request.
