# Supplementary material for: Human-built environment interactions: the relationship between subjective well-being and perceived neighborhood environment characteristics
Source: Sci Rep. 2022 Dec 17;12:21844. doi: 10.1038/s41598-022-25414-9 (PMC9759554; doi:10.1038/s41598-022-25414-9)
Supplement: Supplementary file 1 — Supplementary Information 1. [file 41598_2022_25414_MOESM1_ESM.docx]

**Appendix 1:** The Questionnaire

| **Demographic Characteristics** | | |
| --- | --- | --- |
| 🞏 Male 🞏 Female | Gender: | P.1 |
| 🞏 18-25 🞏 26-35 🞏 36-45 🞏 46-55 🞏 56-65 🞏 66 and more | Age: | P.2 |
| 🞏 Single 🞏 Married 🞏 Divorced 🞏 Widow | Marital Status: | P.3 |
| 🞏 Under diploma 🞏 Diploma 🞏 Associate degree 🞏 Bachelor’s degree 🞏 Master’s degree and higher | Educational Status: | P.4 |
| 🞏 Unemployed or having a non-fixed job 🞏 Student 🞏 Part-time job 🞏 Full-time job 🞏 Housewife 🞏 Retired | Occupation Status: | P.5 |
| 🞏 I live alone 🞏 I live with my family 🞏 I live with my friends 🞏 I live in residence halls | Living Conditions: | P.6 |
| 🞏 Good 🞏 Normal 🞏 Bad | Relationship with family: | P.7 |
| 🞏 Good 🞏 Normal 🞏 Bad | Living Level: | P.8 |
| 🞏 We own this apartment 🞏 It is a rental apartment | Housing ownership status: | P.9 |
| 🞏 less than 1500$ 🞏 1501-2500$ 🞏 2501-4000$ 🞏 more than 4000$ | Monthly Income of Family: | P.10 |
| 🞏 Yes 🞏 No | Do you volunteer or work regularly with NGOs? | P.11 |
| 🞏 Yes 🞏 No | Do you consider yourself an influential and useful person for society? | P.12 |
| 🞏 Once at most 🞏 One or two times 🞏 Two to five times 🞏 More than five times 🞏 More than these | How many times a week do you come to this neighborhood for any reason? | P.13 |
| 🞏 5 minutes at most 🞏 5 to 30 minutes 🞏 30 minutes to 2 hours 🞏 More than 2 hours  🞏 More than these | How much time per week do you spend talking with friends and acquaintances in this neighborhood? | P.14 |
| 🞏 Less than one year 🞏 Between one and two years 🞏 Between three and five years 🞏 More than five years 🞏 More than these | How long have you known this neighborhood and go there? | P.15 |
| 🞏 I live here. 🞏 I work here. 🞏 I cross this neighborhood. 🞏 Shopping 🞏 Restaurants 🞏 Recreation and fun 🞏 Walking and exercise. 🞏 Meet friends 🞏 daily activities  🞏 Others: …………… | What is the motivation for your presence and the type of activities you do in this neighborhood? | P. 16 |

| **A) Environmental Characteristics of The Neighborhood** | | | | | | |
| --- | --- | --- | --- | --- | --- | --- |
| Num. | Questions | Strongly  Disagree | Disagree | Neutral | Agree | Strongly  Agree |
| A.1 | I believe that a Diversity of activities such as (stores, supermarkets, post offices, schools, fast foods, restaurants, banks, etc.) can be seen in this neighborhood. |  |  |  |  |  |
| A.2 | I believe I can easily access public transportation such as the subway, bus, and taxi in this neighborhood. |  |  |  |  |  |
| A.3 | I believe that the distance between my place of residence and my workplace is suitable. |  |  |  |  |  |
| A.4 | I believe that the streets are connected in this neighborhood, and I can easily walk from one street to another. |  |  |  |  |  |
| A.5 | I believe that the sidewalks in this neighborhood have Desirable Pavement. |  |  |  |  |  |
| A.6 | I believe that the lighting is appropriately provided in this neighborhood. |  |  |  |  |  |
| A.7 | I believe there is enough furniture for people to sit in this neighborhood. |  |  |  |  |  |
| A.8 | I believe there are many green and open spaces in this neighborhood. |  |  |  |  |  |
| A.9 | I believe that the form of buildings in this neighborhood is attractive and beautiful. |  |  |  |  |  |
| A.10 | I believe there are places for people's social cohesion in this neighborhood (parks, green spaces, cultural centers, etc.) |  |  |  |  |  |
| A.11 | I believe the number of residential buildings in this neighborhood is balanced. |  |  |  |  |  |
| A.12 | I believe that there is not much crime in this neighborhood. |  |  |  |  |  |

| **B) The Negative and Positive Affect Scale (Negative Affect 1-6; Positive Affect 7-12.)** | | | | | | |
| --- | --- | --- | --- | --- | --- | --- |
| Num. | Questions | Strongly  Disagree | Disagree | Neutral | Agree | Strongly  Agree |
| B.1 | I often feel sad, and nothing can cheer me up. |  |  |  |  |  |
| B.2 | I feel nervous most of the time. |  |  |  |  |  |
| B.3 | I feel restless or fidgety most of the time. |  |  |  |  |  |
| B.4 | I feel hopeless most of the time. |  |  |  |  |  |
| B.5 | I feel struggling most of the time. |  |  |  |  |  |
| B.6 | I feel worthless most of the time. |  |  |  |  |  |
| B.7 | I feel cheerful most of the time. |  |  |  |  |  |
| B.8 | I feel like I’m in good spirits most of the time. |  |  |  |  |  |
| B.9 | I feel extremely happy most of the time. |  |  |  |  |  |
| B.10 | I feel calm and peaceful most of the time. |  |  |  |  |  |
| B.11 | I feel satisfied most of the time. |  |  |  |  |  |
| B.12 | I feel sad most of the time. |  |  |  |  |  |

| **C) Life Satisfaction Scale** | | | | | | |
| --- | --- | --- | --- | --- | --- | --- |
| Num. | Questions | Strongly  Disagree | Disagree | Neutral | Agree | Strongly  Agree |
| C.1 | In most ways, my life is close to my ideal. |  |  |  |  |  |
| C.2 | The conditions of my life are excellent |  |  |  |  |  |
| C.3 | I am satisfied with my life |  |  |  |  |  |
| C.4 | So far, I have gotten the important things I want in life |  |  |  |  |  |
| C.5 | If I could live my life over, I would change almost nothing. |  |  |  |  |  |

| **D) Mental Well-being Scale** | | | | | | |
| --- | --- | --- | --- | --- | --- | --- |
| Num. | Questions | Strongly  Disagree | Disagree | Neutral | Agree | Strongly  Agree |
| D.1 | I’ve been feeling optimistic about the future |  |  |  |  |  |
| D.2 | I’ve been feeling useful |  |  |  |  |  |
| D.3 | I’ve been feeling relaxed |  |  |  |  |  |
| D.4 | I’ve been Dealing with problems well |  |  |  |  |  |
| D.5 | I’ve been thinking clearly |  |  |  |  |  |
| D.6 | I’ve been Feeling close to other people |  |  |  |  |  |
| D.7 | I’ve been able to make up my mind about things. |  |  |  |  |  |

| **E) “Feeling of happiness” Scale** | | | | | | |
| --- | --- | --- | --- | --- | --- | --- |
| Num. | Questions | Strongly  Disagree | Disagree | Neutral | Agree | Strongly  Agree |
| E.1 | Consider myself: not a very happy or unhappy person |  |  |  |  |  |
| E.2 | Compared to most of my peers, I consider myself: less happy or happier |  |  |  |  |  |
| E.3 | Some people are generally very happy. They enjoy life regardless of what is going on. To what extent does this characterization describe you? |  |  |  |  |  |
| E.4 | Some people are generally not very happy. Although they are not depressed, they never seem as happy as they might be. To what extent does this characterization describe you?” |  |  |  |  |  |

| **F) Social inclusion** | | | | | | |
| --- | --- | --- | --- | --- | --- | --- |
| Num. | Questions | Strongly  Disagree | Disagree | Neutral | Agree | Strongly  Agree |
| F.1 | My income status is good throughout the year. |  |  |  |  |  |
| F.2 | I work full time. |  |  |  |  |  |
| F.3 | I participate in neighborhood social activities. |  |  |  |  |  |
| F.4 | I do activities such as sports and visiting libraries, and other activities that entertain me. |  |  |  |  |  |
| F.5 | When needed, I receive social help (from family, friends, and neighbors). |  |  |  |  |  |

| **G) Mental and Physical Health Scale** | | | | | | |
| --- | --- | --- | --- | --- | --- | --- |
| Num. | Questions | Strongly  Disagree | Disagree | Neutral | Agree | Strongly  Agree |
| G.1 | In general, in terms of health, I have excellent conditions. |  |  |  |  |  |
| G.2 | During the day, I usually do light physical activities (such as moving a table, pushing a vacuum cleaner, and light sports). |  |  |  |  |  |
| G.3 | During the day, I may continuously go up and down several flights of stairs. |  |  |  |  |  |
| G.4 | During the last month, I had no problem doing my activities |  |  |  |  |  |
| G.5 | In the previous month, I had no mental problems, including feelings of depression and anxiety, to carry out my activities. |  |  |  |  |  |
| G.6 | During the last month, the feeling of pain and discomfort made it difficult to do my activities both at home and at work. |  |  |  |  |  |
| G.7 | Over the past month, I have been feeling calm and peaceful. |  |  |  |  |  |
| G.8 | I have been full of energy for the past month. |  |  |  |  |  |
| G.9 | During the last month, I have been feeling downhearted and blue |  |  |  |  |  |
| G.10 | During the past month, physical problems have caused disruptions in my social activities (such as visiting friends, relatives, etc.). |  |  |  |  |  |
| G.11 | During the last month, emotional problems have disrupted my social activities (such as visiting friends, relatives, etc.) |  |  |  |  |  |
