# Supplementary material for: Human-built environment interactions: the relationship between subjective well-being and perceived neighborhood environment characteristics
Source: Sci Rep. 2022 Dec 17;12:21844. doi: 10.1038/s41598-022-25414-9 (PMC9759554; doi:10.1038/s41598-022-25414-9)
Supplement: Supplementary file 2 — Supplementary Information 2. [file 41598_2022_25414_MOESM2_ESM.docx]

**Appendix 2:** Frequency of respondents' agreement with each item

|  | **Item** | **Frequency** | | | | |
| --- | --- | --- | --- | --- | --- | --- |
|  |  | **Totally disagree** | **Disagree** | **No idea** | **Agree** | **Totally agree** |
| **A) Environmental characteristics of the neighborhood** | | | | | | |
| A1 | I believe that a Diversity of activities such as (store, supermarket, post office, school, fast food, restaurant, bank, etc.) can be seen in this neighborhood? | 7 | 21 | 8 | 42 | 19 |
| A2 | I believe that in this neighborhood I can easily access public transportation such as (metro, bus, taxi)? | 11 | 27 | 6 | 32 | 21 |
| A3 | I believe that the distance between my place of residence and my workplace is suitable? | 8 | 15 | 14 | 40 | 20 |
| A4 | I believe that in this neighborhood the streets are connected to each other and I can easily walk from one street to another? | 3 | 16 | 5 | 41 | 32 |
| A5 | I believe that the sidewalks in this neighborhood have Desirable Pavement? | 43 | 41 | 10 | 2 | 1 |
| A6 | I believe that the lighting is properly provided in this neighborhood? | 44 | 43 | 6 | 4 | 0 |
| A7 | I believe that there is enough furniture for people to sit in this neighborhood? | 48 | 42 | 3 | 2 | 2 |
| A8 | I believe there are many green and open spaces in this neighborhood? | 55 | 35 | 5 | 2 | 0 |
| A9 | I believe that the form of buildings in this neighborhood is attractive and beautiful? | 53 | 37 | 4 | 2 | 1 |
| A10 | I believe that there are places for people's social cohesion in this neighborhood (parks, green spaces, cultural centers, etc.) | 50 | 41 | 4 | 2 | 0 |
| A11 | I believe that the number of residential buildings in this neighborhood is balanced? | 7 | 13 | 26 | 41 | 10 |
| A12 | I believe that there is not much crime in this neighborhood? | 45 | 38 | 10 | 4 | 0 |
| **B) The negative and positive effect** | | | | | | |
| B1 | I often feel sad, and nothing can cheer me up. | 12 | 18 | 15 | 31 | 21 |
| B2 | I feel nervous most of the time. | 10 | 25 | 12 | 25 | 25 |
| B3 | I feel restless or fidgety most of the time. | 6 | 25 | 8 | 25 | 33 |
| B4 | I feel hopeless most of the time. | 13 | 28 | 14 | 21 | 21 |
| B5 | I feel struggling most of the time. | 2 | 11 | 7 | 47 | 30 |
| B6 | I feel worthless most of the time. | 30 | 31 | 7 | 23 | 6 |
| B7 | I feel cheerful most of the time. | 8 | 47 | 9 | 25 | 8 |
| B8 | I feel like I’m in good spirits most of the time. | 7 | 45 | 10 | 27 | 8 |
| B9 | I feel extremely happy most of the time. | 8 | 46 | 19 | 15 | 9 |
| B10 | I feel calm and peaceful most of the time. | 12 | 42 | 20 | 15 | 8 |
| B11 | I feel satisfied most of the time. | 14 | 40 | 18 | 18 | 7 |
| B12 | I feel sad most of the time. | 13 | 45 | 14 | 16 | 9 |
| **C)** **Life Satisfaction Scale** | | | | | | |
| C1 | In most ways, my life is close to my ideal. | 29 | 30 | 10 | 25 | 3 |
| C2 | The conditions of my life are excellent | 22 | 35 | 15 | 20 | 5 |
| C3 | I am satisfied with my life | 16 | 36 | 9 | 30 | 6 |
| C4 | So far, I have gotten the important things I want in life | 23 | 20 | 18 | 15 | 21 |
| C5 | If I could live my life over, I would change almost nothing. | 47 | 24 | 16 | 4 | 6 |
| **D) Mental Well-being Scale** | | | | | | |
| D1 | I’ve been feeling optimistic about the future | 19 | 19 | 12 | 26 | 21 |
| D2 | I’ve been feeling useful | 12 | 16 | 20 | 33 | 16 |
| D3 | I’ve been feeling relaxed | 13 | 30 | 16 | 26 | 12 |
| D4 | I’ve been Dealing with problems well | 5 | 17 | 18 | 44 | 13 |
| D5 | I’ve been thinking clearly | 7 | 15 | 26 | 42 | 7 |
| D6 | I’ve been Feeling close to other people | 8 | 14 | 19 | 37 | 19 |
| D7 | I’ve been able to make up my mind about things. | 9 | 13 | 17 | 45 | 13 |
| **E) “Feeling of Happiness” Scale** | | | | | | |
| E1 | Consider myself: not a very happy or unhappy person | 12 | 30 | 13 | 22 | 20 |
| E2 | Compared to most of my peers, I consider myself: less happy or happier | 17 | 32 | 13 | 17 | 18 |
| E3 | Some people are generally very happy. They enjoy life regardless of what is going on. To what extent does this characterization describe you? | 16 | 27 | 9 | 26 | 19 |
| E4 | Some people are generally not very happy. Although they are not depressed, they never seem as happy as they might be. To what extent does this characterization describe you?” | 9 | 15 | 14 | 33 | 26 |
| **F) Social Inclusion** | | | | | | |
| F1 | My income status is good throughout the year. | 24 | 24 | 15 | 22 | 12 |
| F2 | I work full time. | 17 | 15 | 18 | 27 | 20 |
| F3 | I participate in neighborhood social activities. | 32 | 29 | 4 | 1 | 31 |
| F4 | I do activities such as sports and visiting libraries, and other activities that entertain me. | 6 | 10 | 4 | 11 | 66 |
| F5 | When needed, I receive social help (from family, friends, and neighbors). | 3 | 8 | 4 | 12 | 70 |
| **G) Mental and Physical Health Scale** | | | | | | |
| G1 | In general, in terms of health, I have excellent conditions. | 2 | 2 | 8 | 10 | 75 |
| G2 | During the day, I usually do light physical activities (such as moving a table, pushing a vacuum cleaner, and light sports). | 2 | 4 | 4 | 13 | 74 |
| G3 | During the day, I may continuously go up and down several flights of stairs. | 4 | 12 | 2 | 39 | 40 |
| G4 | During the last month, I had no problem doing my activities | 8 | 14 | 5 | 38 | 32 |
| G5 | In the previous month, I had no mental problems, including feelings of depression and anxiety, to carry out my activities. | 12 | 15 | 10 | 37 | 23 |
| G6 | During the last month, the feeling of pain and discomfort made it difficult to do my activities both at home and at work. | 20 | 36 | 15 | 19 | 7 |
| G7 | Over the past month, I have been feeling calm and peaceful. | 18 | 26 | 33 | 15 | 5 |
| G8 | I have been full of energy for the past month. | 16 | 27 | 21 | 26 | 7 |
| G9 | During the last month, I have been feeling downhearted and blue | 14 | 20 | 14 | 36 | 13 |
| G10 | During the past month, physical problems have caused disruptions in my social activities (such as visiting friends, relatives, etc.). | 36 | 28 | 6 | 5 | 22 |
| G11 | During the last month, emotional problems have disrupted my social activities (such as visiting friends, relatives, etc.) | 36 | 31 | 6 | 18 | 6 |

**Table 1. Frequency of Items related to Environmental Characteristics of the Neighborhood**

**Table 2. Frequency of Items related to the Negative and Positive Effect**

**Table 3. Frequency of Items related to Life Satisfaction Scale**

**Table 4. Frequency of Items related to Mental Well-being Scale**

**Table 5. Frequency of Items related to “Feeling of Happiness” Scale**

**Table 6. Frequency of Items related to Social Inclusion**

**Table 7. Frequency of Items related to Mental and Physical Health Scale**
